# Epidemiology and Management of Acute Hematogenous Osteomyelitis, Neonatal Osteomyelitis and Spondylodiscitis in a Third Level Paediatric Center

**DOI:** 10.3390/children8080616

**Published:** 2021-07-21

**Authors:** Paola Musso, Sara Parigi, Grazia Bossi, Gian Luigi Marseglia, Luisa Galli, Elena Chiappini

**Affiliations:** 1 Pediatric Clinic, Department of Pediatrics, Fondazione IRCCS Policlinico San Matteo, University of Pavia, 27100 Pavia, Italy; g.bossi@smatteo.pv.it (G.B.); gl.marseglia@smatteo.pv.it (G.L.M.); 2 Department of Health Sciences, University of Florence, 50100 Florence, Italy; saraparigi91@gmail.com (S.P.); luisa.galli@unifi.it (L.G.); elena.chiappini@unifi.it (E.C.)

**Keywords:** osteomyelitis, spondylodiscitis, newborn, bone infection, children

## Abstract

Acute hematogenous osteomyelitis (AHOM) is a rare pathology in pediatric population. The aim of this study is to analyse the epidemiological data and the management, compared to European Society for Paediatric Infectious Disease (ESPID, European Society for Pediatric Infectious Diseases) guidelines 2017 of 216 children with AHOM, divided in three cohorts (neonatal-onset osteomyelitis, those with vertebral involvement and other types of osteomyelitis). We conducted a retrospective single center study, evaluating data from all the children (aged 0–18 years) consecutively admitted to the Meyer Children’s Hospital, during a period of ten years (1 January 2010–31 December 2019). Isolation of pathogen was possible in 65 patients and *S. aureus* was the most frequently involved (43/65 children). Magnetic Resonance Imaging (MRI, magnetic resonance imaging) was performed in 201/216 cases and was compatible with osteomyelitis in 185/201 of these children (92.03%). In the neonatal-onset osteomyelitis group the percentage of diagnostic ultrasound for osteomyelitis was 36.36% significantly higher than the other groups. The median duration of total antibiotic therapy was 37.5 days. In total, 186/199 children recovered without complications. The present study delineates three heterogeneous cohorts of patients. *S. aureus* is confirmed as the first pathogen for isolation in all three groups analysed. MRI represent a gold standard for diagnosis. Longer duration of antibiotics treatment was performed in neonatal and spondylodiscitis group, compared to the other types of osteomyelitis.

## 1. Introduction

Osteomyelitis is a rare pathology in the pediatric population: the incidence is higher in males and the mean age of onset is around five years [1,2,3,4,5]. The sites most involved are the metaphysis of the lower bones [6].

The microorganisms most frequently implicated in AHOM are Gram positive bacteria in most cases, followed by Gram negative bacteria and less frequently mycobacteria and fungal agents [1,3,5,7,8,9].

The beginning of antibiotic therapy must always be preceded by microbiological investigations to try to isolate the pathogen with cultures or new methods such as polymerase chain reaction (PCR) [10]. Microbiological isolation is fundamental to adapt the antibiotic therapy to the antibiogram [1].

Magnetic resonance imaging (MRI) represents the radiological technique that allows the greatest collection of diagnostic information for osteomyelitis as it is able to show anomalies already after three to five days: it reveals details of the bone and soft tissue involvement, including the formation of abscesses, sequestra or associated pyomyositis or contiguous venous thrombosis [6]. Radiograph imaging, in comparation, is frequently normal at baseline and reveal alterations mostly 10–21 days after onset of symptoms [6].

The ESPID guidelines (European Society for Pediatric Infectious Diseases) recommend different empirical intravenous antibiotic therapy schemes depending on age: in children aged < 3 months the combination of cefazolin or an anti-staphylococcal penicillin with gentamicin; monotherapy with a cefazolin or cefuroxime or an anti-staphylococcal penicillin in children ≥ 3 months of age [6].

In cases with strong clinical suspicion of MRSA (*S. aureus* methicillin resistant) osteomyelitis or if the local prevalence of MRSA > 10%, is suggested the empirical use of [11]:

− clindamycin if the prevalence of MRSA is >10% and clindamycin resistance is< 10%;
− vancomycin if the prevalence of MRSA > 10% and clindamycin resistance > 10%.

The duration to switch from intravenous to oral therapy is still a topic under study, in particular clinical criteria such as apyrexia, compliance with oral therapy, pain reduction, local clinical improvement and the general conditions and laboratory criteria (the reduction of indices such as protein C-reactive, erythrocyte sedimentation rate and leukocyte count) were examined [1,11,12,13,14,15,16,17,18].

The 2017 ESPID guidelines recommend switching to oral therapy after 2–4 days of intravenous antibiotic therapy if the patient presents an improvement in clinical conditions: apyrexia or decrease in body temperature for 24–48 h, improvement of symptoms, lack of signs referable to complications, 30–50% decrease in CRP (protein C-reactive) compared to the maximum value reached, negative culture tests and the absence of pathogens such as MRSA or PVL-SA (Panton-Valentine leucocidin positive *S. aureus*) that can cause more severe forms of osteomyelitis [6].

Oral therapy in uncomplicated acute hematogenous osteomyelitis should be continued for 3–4 weeks, carefully monitoring the markers of inflammation. Oral therapy can be continued at home, allowing for discharge and follow up [6].

In case of isolation of *S. aureus* MRSA or PVL +, aged < 3 months, inadequate response or complicated osteomyelitis or vertebral or pelvic involvement the 2017 ESPID guidelines recommend continuing therapy for a total of 4–6 weeks [6]. A total of 90% of pediatric patients with acute hematogenous osteomyelitis can only be treated with antibiotic therapy, particularly if antibiotic therapy is started during the first days of symptom onset [19,20,21].

In neonatal osteomyelitis, the antibiotics to be used must be effective against pathogens such as *S. aureus*, Group B Streptococcus and Gram negative bacteria of the gastrointestinal tract [22].

Empiric antibiotic therapy must include an antistaphylococcal molecule (nafcillin or oxacillin) associated with a second or third generation cephalosporin (cefotaxime, ceftriaxone, ceftazidime) or an aminoglycoside (gentamicin, amikacin, tobramycin) to allow broad coverage against the main pathogens involved. In the case of neonates at high risk for nosocomial pathogens (*S. aureus* MRSA or coagulase negative), nafcillin should be replaced by a glycopeptide (vancomycin, teicoplanin) [22,23,24].

The aim of this study was to retrospectively analyse the epidemiological data and the management of a cohort of children with acute hematogenous osteomyelitis afferent to a pediatric third-level center over a period of ten years, with particular attention to the subgroups of children with neonatal-onset osteomyelitis and those with vertebral involvement.

## 2. Materials and Methods

We conducted a retrospective single center study, evaluating data from all the children (aged between 0 month and 18 years) consecutively admitted to the Meyer Children’s Hospital with a World Health Organization International Classification of Diseases (WHO ICD−10) discharge code for osteomyelitis, between 1 January 2010 and 31 December 2019.

Exclusion criteria were congenital or acquired immunodeficiency, underlying bone diseases, carriers of prosthetic materials; those with hospital acquired infections, infection secondary to significant trauma, isolated septic arthritis (i.e., no adjacent osteomyelitis on imaging). Demographic and clinical details, microbiological and radiologic results, and clinical management including type, route and duration of antibiotic treatment, need for surgery were anonymously entered into an electronic database.

Meyer Children’s University Hospital is a tertiary paediatric hospital with 200 paediatric beds and serves as the main referral center for Tuscany and surrounding regions.

All the laboratory tests were performed in the same laboratory at the Meyer Children’s Hospital, using standardized techniques and according to manufacturer’s instructions.

Haematological parameters such as WBC (white blood count), CRP (protein C-reactive), and ESR (erythrocyte sedimentation rate) were collected at admission before administration of any antimicrobial therapy. In particular, CRP in serum or heparinized plasma was detected by means of particle enhanced immunonephelometry on the Dimension VistaTM System (Dimension Vista™ System Flex^®^ reagent cartridge, Siemens Healthcare Diagnostics Inc, Newark, DE, USA). The analytical measurement range (AMR) was 0.29–19.0 mg/dL.

ESR was measured using a capillary micro-photometer method and expressed in mm/h. The AMR is 2–120 mm/h.

Blood cultures and cultures from bone or joint fluid samples were processed using standard methods: all samples were processed for detection of common human bacteria (or cultured for detection of MRSA isolates) using rich and selective culture media. After 48 h of incubation in aerobic atmosphere at 37 °C, plates were read in order to detect the presence of pathogens. Identification of bacteria isolates were based on typical colony morphology on selective culture media (Mannitol Salt 2 Agar, Mac Conkey Agar, bioMérieux, Florence, Italy) or on chromogenic culture media (chromID^®^ CARBA SMART, bioMérieux, Florence, Italy, and BBL™ CHROMagar™ MRSA II, Becton Dickinson, Franklin Lakes, NJ, USA). In order to confirm species-level identification mass spectrometry analysis was performed using MALDI-TOF (VITEK^®^ MS, bioMérieux, Florence, Italy). Organisms were identified phenotypically and confirmed using traditional methods or the Vitek2 gram positive card (bioMerieux, Florence, Italy).

Universal real-time polymerase chain reaction assay (PCR) targeting the gene coding for 16S ribosomal RNA coupled with sequencing of amplified products was performed on blood and/or bone/joint samples.

Data were reported as median and interquartile range (IQR) or absolute numbers and percentages. All continuous variables were not normally distributed, thus the non parametric Kruskal–Wallis test was used to compare groups. Fisher’s exact test or Chi square test were used to compare categorical variables, as appropriate. A *p* value < 0.05 was considered significant.

All statistical analyses were carried out using the SPSS (Statistical Package of Social Sciences, Chicago, IL, USA) for Windows software program version 19.0.

## 3. Results

Two hundred and sixteen children, diagnosed with AHOM, were included in the study. Among these, 12 patients presented an onset within 3 months of birth, 22 presented vertebral involvement and 182 presented osteomyelitis of bones different to vertebrae with age over 3 months: this cohort will be called in text; other types of osteomyelitis. The characteristics of the included children are shown in Table 1.

The median patient’s age of diagnosis was 5 years (IQR 1–11) for spondylodiscitis, 35 days of life (IQR 24–50) for neonatal osteomyelitis and 5 years (IQR 1–10) for other types of osteomyelitis. Among the 216 children, 128 were male (59.26%) and the male prevalence is confirmed in all the three groups of patients.

The 54.21% of children (116/216) presented with fever at the time of admission; in particular, in the group of other types of osteomyelitis the 57.78% of patients presented with fever at admission, while among the groups of neonatal disease and spondylodiscitis, the majority of patients were apyretic at admission (*p* = 0.047).

Most of children presented one or more signs of local inflammation, in particular swelling (119/216, 55.87%), pain (186/216, 86.92%) and movement limitation (160/216, 75.12%) at diagnosis, but with statistically significant differences within the three groups: in spondylodiscitis swelling, redness and warm were not found at diagnosis, while pain (16/22, 72.73%) and movement limitation (16/22, 72.73%) were markedly more expressed compared to the other signs of inflammation; in children affected by other types of osteomyelitis, pain (136/182, 90.56%) prevailed over other signs of inflammation. Anyway, the majority of these children also presented warm and swelling in a percentage greater than 50% (respectively, 54.19% and 60.89%). In neonatal onset osteomyelitis, swelling (9/12, 75.00%) was the most common sign; however, warm (50.00%), pain (58.33%) and redness (50.00%) occurred in the majority of patients.

The median time interval between the onset of symptoms and the start of antibiotic therapy was 7.0 days (IQR 3.0–13.0) with statistically significant differences within the three groups. Spondylodiscitis affected children had a median interval of 12 days IQR (7–27), greater than the group of other osteomyelitis (7 days, IQR 3–13, *p* = 0.002) and the neonatal-onset osteomyelitis (2 days IQR 2–7, *p* < 0.001).

In other-type osteomyelitis and neonatal-onset osteomyelitis, the lower limb bones were the mostly affected (111/194, 57.21%). Long bones were affected in the majority of cases (111/194, 57.21%). In particular foot (39/194, 20.10%), femur (33/194, 17.01%), tibia (32/194 16.49%) and fibula (7/194, 3.61%), followed by the upper limb bones: humerus (23/194, 11.86%), ulna (4/194, 2.06%), radius 3/194, 1.55%). Long bones were affected in the majority of cases (111/194, 57.21%).

The metaphysis was involved in 84/119 cases (70.58%), the epiphysis in 77/119 cases (64.70%), while diaphyseal involvement was observed in 43/119 cases (36.13%).

In neonatal-onset osteomyelitis group 1/12 patients (8.33%) had a multifocal bones involvement, in the spondylodiscitis group, the most involved vertebral segment was the lumbar (7/21, 31.81%), followed by the sacral one (4/21, 18.18%).

One hundred fifteen of 216 children (53.24%) presented complicated AHOM; a statistically significant difference was observed in the neonatal-onset osteomyelitis group, with 66.67% (8/12) complicated disease (*p* = 0.008).

Is defined complicated AHOM a osteomyelitis that presents at least one of the characteristics included in the Table 2: sepsis, septic shock, arthritis, cellulitis, subperiosteal abscess, muscular abscess, deep vein thrombosis, fracture, septic emboli or ICU admission. (Table 2).

A statistically significant difference was found across the three groups regarding arthritis (*p* = 0.039), subperiosteal abscess (*p* = 0.019) and ICU admission (*p* = 0.002). Arthritis is almost never present in spondylodiscitis (1/12, 4.55%) compared to the other type osteomyelitis (54/182, 29.67%) and neonatal-onset osteomyelitis (4/12, 33.33%); the subperiosteal abscess represents a major complication in the neonatal-onset group of osteomyelitis (5/12, 41.67%) compared to that of other osteomyelitis (29/182, 15.93%) or spondylodiscitis (1/22, 4.55%); ICU admission was necessary in 25.00% of patients with neonatal-onset osteomyelitis (3/12) compared to 3.30% of patients with other type osteomyelitis and 4.55% of patients with spondylodiscitis.

Table 3 show values at diagnosis of white blood cell (WBC), erythrocytes sedimentation rate (ESR) and C-reactive protein (CRP) of three groups of patients with AHOM. On 216 patients only for 205 was possible recover laboratory exams performed before the start of antibiotic therapy.

Normal WBC count (<12,000/mm^3^) was observed in 132/205 children (64.39%); of these, 17/21 (80.95%) were affected by spondylodiscitis, 4/12 (33.33%) by neonatal-onset osteomyelitis and 111/164 (64.53%) by osteomyelitis of another type (*p* = 0.023). Normal ESR values (<15 mm/h) were observed in 23/205 (13.45%) children; no child with spondylodiscitis had negative ESR values at diagnosis. Normal CRP (<0.5 mg/dL) were observed in 46/205 (22.12%) children; 4/21 (22.12%) with spondylodiscitis, 3/12 (25.00%) with neonatal osteomyelitis and 39/172 (22.29%) with osteomyelitis of another type.

Krustal–Wallis statistical test on the median values of WBC at diagnosis did not show statistically significant differences between the three groups of patients.

Overall, 156/216 (72.22%) children were subjected to at least one microbiological test: blood culture was performed in 85/216 (39.35%) children and was positive in 37/85 (43.52%) of cases, culture on pus or biopsy was performed in 53/216 (24.54%) cases with isolation of a pathogen in 26/53 (49.05%) cases. PCR real-time on blood sample was performed in 119/216 (55.09%) cases and was positive in 8/119 (6.72%). PCR real-time on pus/biopsy sample was performed in 55/216 (25.46%) children and resulted positive in 33/55 (60%).

At least one pathogen was isolated in 65/216 cases (30.09%), of which 54/182 (29.67%) with other type osteomyelitis, 6/12 (50.00%) with neonatal-onset osteomyelitis and 5/22 (22.73%) with spondylodiscitis.

*S. aureus* was the most frequently involved pathogen, isolated in 43/65 (66.15%) children of whom microbiological isolation was possible. It was isolated in 29/37 (78.37%) positive blood cultures, 22/33 (66.66%) PCR positive pus or biopsy, 15/53 (28.30%) positive pus or biopsy cultures, and 1/8 (12.5%) positive PCR on blood. Analysing the three cohorts of children, *S. aureus* was isolated in 37/182 (20.32%) patients with other type osteomyelitis, 3/12 (25.00%) patients with neonatal-onset osteomyelitis and 3/22 (13.63%) patients with spondylodiscitis. In this latter group of children, the percentage of *S. aureus* isolation was lower than in other groups (*p* = 0.022). Overall, 54/182 (29.67%) children with other type osteomyelitis, 6/12 (50%) patients with neonatal-onset osteomyelitis and 5/22 (22.72%) patients with spondylodiscitis obtained a microbiological isolation.

The other isolated pathogens were: *Streptococcus pyogenes* (4/65, 6.15%), *Pseudomonas aeruginosa* (3/65, 4.61%), *Streptococcus agalactiae* (2/65, 3.07), and *Escherichia coli* (1/65, 1.54%); five children had a polymicrobial isolation (5/65, 7.69%) in particular: *Staphylococcus simulans* and *Corynebacterium amycolatum* (1 child with other type osteomyelitis), *Veilonella parvula* and *Fusobacterium* (1 child with other type osteomyelitis), *S. aureus* and *Streptococcus agalactiae* (1 child with spondylodiscitis), *S.pyogenes* and *E.coli* (1 child with spondylodiscitis), *Streptococcus mitis* and *Rothia mucilaginosa* (1 child with other type osteomyelitis).

All cases with microbiological isolation of *Pseudomonas aeruginosa* (3/65, 4.61%) were found in the other type osteomyelitis group.

More than one radiological exam was performed in all patients during the diagnostic process. Conventional X-rays were performed in 183/216 children (84.72%). In 10.38% of these children (19/183), X-rays result was compatible with osteomyelitis. MRI was performed in 201/216 cases (93.05%) and was compatible with osteomyelitis in 185/201 of these children (92.03%). Computed tomography (CT) was performed in 17/216 children (7.87%) and findings consistent with the diagnosis of osteomyelitis were observed in 10/17 cases (58.82%). Bone scan was performed in 8/216 children (3.70%), detecting results compatible with osteomyelitis in 7/8 cases (87.5%). Ultrasound (US) was performed in 124/216 children (57.40%), highlighting findings compatible with osteomyelitis in only 15/124 children (12.09%). However, in the neonatal-onset osteomyelitis group the percentage of diagnostic US for osteomyelitis was 36.36% (4/11) significantly higher than the other groups (*p* = 0.011).

The median duration of total antibiotic therapy (intravenous and oral) was 37.5 days (IQR 29–48): other type osteomyelitis received a median total duration of 37 days (IQR 28–46), neonatal-onset osteomyelitis 48 days (IQR 41–70), and the spondylodiscitis group 48 days (IQR 34–70).

The Krustal–Wallis statistical test on the median duration of total antibiotic therapy showed a significantly longer duration in neonatal-onset osteomyelitis (48 days, IQR 41–70) and spondylodiscitis (48 days IQR 34–70), when compared to the other type osteomyelitis group of children (37 days IQR 28–46, *p* = 0.001).

All 216 included patients received intravenous therapy at admission for median 21 days (IQR 15.5–28.5): other type osteomyelitis group received intravenous therapy with a median duration of 21 days IQR (15–27), neonatal-onset osteomyelitis group of 33 days (IQR 27–44), while the spondylodiscitis group for 25.5 days (IQR 19–32).

The Krustal–Wallis test showed a significantly difference of duration of intravenous therapy between the group of other type osteomyelitis (21 days IQR 15–27) and that of osteomyelitis with neonatal onset (33 days IQR 27–44, *p* < 0.001). The median duration of oral antibiotic therapy was 15 days (IQR 13–30) and no statistically significant difference was found among the three group of children. (Table 4).

The intravenous first-line treatment most frequently used was the combination of antistaphylococcal penicillin together with third generation cephalosporin (129/209 cases, 61.72%), followed by glycopeptide associated with third generation cephalosporin (13/209 cases; 22%), oxacillin monotherapy (11/209; 5.26%), clindamycin associated with third generation cephalosporin (9/209 cases; 4.31%), cephalosporin monotherapy (6/209; 2.87%) and the clindamycin monotherapy (4/209, 1.91%). In the neonatal-onset osteomyelitis group, the combination of ampicillin and aminoglycoside was more frequently used than in the other groups (4/12 cases, 36.36%, *p* < 0.001).

The reason to start with different empirical antibiotics regimes depends to the age of patients (ampicillin plus aminoglycoside is more frequently used in neonatal group compared to others) and the severity of clinical condition: the use of glycopeptide or clindamycin instead of antistaphylococcal penicillin (in association with the third generation cephalosporin) was reserved to children with more severe condition at diagnosis based on clinical medical opinion; oxacillin or cephalosporin in monotherapy was chosen for patients with less severe clinical conditions at diagnosis.

In 102/204 cases (50.00%), first-line intravenous therapy was switched to a second-line antibiotic treatment, and, in particular, in 40 cases (39.22%) to a glycopeptide in monotherapy, in 8 cases (7.84%) to a glycopeptide associated with third generation cephalosporin, in 5 cases (4.90%) to clindamycin alone and in 5 cases (4.90%) first-line therapy was modified to meropenem associated with a glycopeptide.

Table 5 shows first line intravenous antibiotic therapy used in the three groups of children.

In 102/204 cases (50.00%), first-line intravenous therapy was switched to a second-line antibiotic treatment, and in 40 cases (39.22%) to a glycopeptide in monotherapy. In 176/206 cases (85.44%) intravenous therapy was switched to oral therapy and the most frequently used drug was amoxicillin-clavulanate (95/206; 53.98%) (Table 6).

One hundred and eighty-six children out of 199 (93.47%) fully recovered. Among these, 156/167 were affected by other types of osteomyelitis (93.41%), 11/12 by neonatal-onset osteomyelitis (91.67%) and 19/20 by spondylodiscitis (95.00%).

About the cases with complicated osteomyelitis, 29/115 (25.21%) underwent a surgical intervention, 86/115 (74.78%) only received antibiotic therapy. In total, 103/115 (89.56%) of them fully recovered, 6/115 (5.22%) recovered but developed movement limitation and 6/115 (5.22%) patients were lost at follow up (5 cases of other types osteomyelitis and 1 of spondylodiscitis).

## 4. Discussion

The present study collected and retrospectively analysed clinical, microbiological and therapeutic data regarding 216 children with AHOM who needed hospitalization in a third level paediatric hospital in Italy, during a period of 10 years.

In line with previous literature, the study confirmed a higher incidence of AHOM in males (59.26%) [1,6,25,26] and in children with a median age of 5 years [2,3,4,5], while the neonatal-onset osteomyelitis cohort showed a median age of 35 days.

The lower limb was most frequently affected, with involvement of long bones (57.21%) such as femur (17.01%) and tibia (16.49%) [7,27]. In spondylodiscitis, the lumbar and sacral vertebrae (72.41%) were the most affected segments [28,29].

The 8% of neonatal cases that had multifocal involvement of bones, in agreement to the literature that report this complication typically for this group of patients [23].

Fever, elevated inflammation indices and movement limitation are frequently observed at onset, but with differences within the three groups analysed: in spondylodiscitis, pain and movement limitation prevailed over other signs of inflammation, while fever was observed in a minority of cases [28]. In neonatal osteomyelitis group, swelling was the prevalent sign of local inflammation, and fever was present only in 41.67% of patients, confirming the importance of local symptoms over systemic ones, such as fever, in neonatal disease [12,27].

The different clinical presentation at onset can determine the different latency time between the symptoms onset and the start of antibiotic therapy: the lower expression of systemic symptom like fever in spondylodiscitis could lead to a greater diagnostic delay in this type of infection (median of 12 days in the present study).

At diagnosis, only 35% of children had leukocytosis (WBC values > 12,000/mm^3^), while ESR and CRP were elevated in majority of patients of all three cohorts. In particular, in spondylodiscitis, no patients presented with negative ESR values at diagnosis. These results are in agreement with previous literature [1,11,12,30].

Among AHOM complications, osteoarthritis and subperiosteal abscesses were the most frequently observed in neonatal forms, in line with previous literature [25], in fact, at birth, metaphyseal vessels penetrate the conjugating cartilage and bone periosteum is more weakly connected to the cortex [31].

MRI showed a sensitivity of 92.04% for the diagnosis of osteomyelitis, as previously described [6,12]. On the contrary, X-ray and ultrasound sensitivities were low (respectively, 10.38% and 12.09%), except for neonatal-onset osteomyelitis group, for whom US was diagnostic in 36.36% of cases, due to the greater incidence of subperiosteal abscesses, detectable on US, in this group of children [32]. This result is worthy of further confirmation and may suggest the use of bedside US in neonatal forms during the diagnostic process [23], even if MRI confirmation remains the gold standard for the diagnosis, as suggest also in literature.

Regarding the microbiological isolation of pathogens, PCR on pus/biopsy showed the highest percentage of isolations (60%), and, overall, a pathogen was isolated in 30.09% of patients.

The present study confirms *S. aureus* as the prevalent aetiological agent (66.15% of cases), in all the three cohorts of children. *Streptococcus pyogenes* and *Streptococcus agalactiae* follow as incidence, in agreement with previous literature [1,6,23], and Gram-negative bacteria (*Escherichia coli* [23,33] and *Pseudomonas aeruginosa*).

In the present study, *Pseudomonas aeruginosa* resulted the most common Gram-negative isolated bacterium.

Median duration of intravenous antibiotic therapy was 21 days (IQR 15.5–28.5) with statistically significant differences between three groups. If compared to ESPID 2017 guidelines [6] the present data showed a longer duration of intravenous antibiotic therapy, up to 33 days of median value for neonatal forms. It was possible to observe a significantly longer duration of intravenous therapy in neonatal-onset osteomyelitis and spondylodiscitis compared to other type osteomyelitis, and these data reflect the paucity of studies (and consequently the lower evidence) concerning these particular forms of osteomyelitis.

As first line intravenous therapy, the combination of anti-staphylococcal penicillin and third generation cephalosporin were the most frequently used (61.72%), followed by glycopeptide and third generation cephalosporin (6.22%). Cephalosporin or anti-staphylococcal penicillin alone (as indicated in the ESPID 2017 guidelines [6]) were used in 9.57% of cases; in particular, oxacillin, ceftriaxone and ceftazidime were the most frequently monotherapy regimens prescribed.

In neonatal-onset osteomyelitis group, 36.36% of patients received the combination of anti-staphylococcal penicillin and third generation cephalosporin (mainly ceftazidime), in line with ESPID 2017 guidelines [6]. Ampicillin + aminoglycoside, which represents the first line therapy for neonatal sepsis, ranked second place.

Regarding the switch to oral therapy, amoxicillin + clavulanic acid was the main prescribed regimen (53.98%), due to its pharmacokinetic and pharmacodynamic profiles and to its efficacy towards MSSA. Overall, 93.47% of patients recovered without complications, while 3.06% reported sequelae.

## 5. Conclusions

The present study compares three pediatric populations affected by AHOM differing for age and site of presentation and delineates three heterogeneous cohorts with different presentation symptom of disease, inflammatory markers levels at diagnosis and duration of antibiotic treatment (Table 7).

In the neonatal group (that included infants younger than three months) it is confirmed the higher incidence of multifocal osteomyelitis; local symptoms prevail over systemic symptoms like fever and swelling is a prevalent sign of local inflammation. In this group, we found greater presence of osteoarthritis and subperiosteal abscesses and higher rate for intensive care admission than in the other groups.

In the relation to the capacity of US to detect subperiosteal abscesses, in the neonatal group, we found more diagnostic exams in comparation to the other groups with statistical difference: this result could suggest to include US if osteomyelitis infection is suspected in neonatal age but MRI remain the gold standard as diagnostic technique.

In the spondylodiscitis group, pain and movement limitation prevail over other symptoms, ESR was elevated in all patients at the time of diagnosis and we found a longer latency time (12 days IQR 2–27) between onset of symptoms and start of antibiotic therapy in comparation to the other groups.

About antibiotic treatment and duration of its prescription we found longer duration of intravenous antibiotic therapy in neonatal and spondylodiscitis groups compared to the group of other types of osteomyelitis and respect of what is indicated in the ESPID guidelines [6].

These results could suggest that more studies are needed on antibiotic management of AHOM in the neonatal age and spine involvement form, to understand if it is possible use a short time with EV therapy and a quick switch to oral therapy as suggest for other type osteomyelitis in the ESPID guidelines [6].

The study also had some limitations. Firstly, it was a retrospective study; secondly, it was not possible to obtain a microbiological isolation in all patients and only 72.77% of patients had a microbiological examination: this aspect have to be improve with realization at least a blood culture in all patients with suspect of AHOM before the started of empiric antibiotic therapy.

## Figures and Tables

**Table 1:** Characteristics of included children.

	Total (*n* = 216)	Other Type Osteomyelitis (*n* = 182)	Neonatal Osteomyelitis (*n* = 12)	Spondylodiscitis (*n* = 22)	Chi-Square	*p* Value
Median patient age (years/days; IQR)		5 years (1–10)	35 days (24–50)	5 years (2–11)		
Sex (*n*, %)						
M	128 (59.26%)	109 (59.89%)	7 (58.33%)	12 (54.55%)	0.2367	0.888
F	88 (40.74%)	73 (40.11%)	5 (41.67%)	10 (45.45%)		
Fever at admission (*n*, %)						
Absent	98 (45.79%)	76 (42.22%)	7 (58.33%)	15 (68.18%)	6.1273	0.047
Present	116 (54.21%)	104 (57.78%)	5 (41.67%)	7 (31.82%)		
Swelling at admission (*n*, %)						
Absent	94 (44.13%)	70 (39.11%)	3 (25.00%)	21 (95.45%)	27.1183	<0.001
Present	119 (55.87%)	109 (60.89%)	9 (75.00%)	1 (4.55%)		
Redness at admission (*n*, %)						
Absent	141 (66.20%)	114 (63.69%)	6 (50.00%)	21 (95.45%)	10.3268	0.006
Present	72 (33.80%)	65 (36.31%)	6 (50.00%)	1 (4.55%)		
Warm at admission (*n*, %)						
Absent	110 (51.64%)	82 (45.81%)	6 (50.00%)	22 (100%)	23.0518	<0.001
Present	103 (48.36%)	97 (54.19%)	6 (50.00%)	0 (0%)		
Pain at admission (*n*, %)						
Absent	28 (13.08%)	17 (9.44%)	5 (41.67%)	6 (27.27%)	14.6120	<0.001
Present	186 (86.92%)	163 (90.56%)	7 (58.33%)	16 (72.73%)		
Movement limitation at admission (*n*, %)						
Absent	53 (24.88%)	43 (24.02%)	4 (33.33%)	6 (27,27)	0.5966	0.742
Present	160 (75.12%)	136 (75.98%)	8 (66.67%)	16 (72.73%)		
Time interval between onset of symptoms and antibiotic therapy days (IQR)	7 (3–13)	7 (3–13)	2 (2–7)	12 (7–27)		0.001

M: Male. F: Female. IQR: interquartile range.

**Table 2:** Acute hematogenous osteomyelitis complications.

	Total (*n* = 216)	Other Type Osteomyelitis (*n* = 184)	Neonatal Osteomyelitis (*n* = 12)	Spondylodis Citis (*n* = 22)	Chi-Square	*p* Value
Osteomyelitis (*n*, %)						
Complicated	115 (53.24%)	102 (56.04%)	8 (66.67%)	5 (22.73%)	9.6714	**0.008**
Sepsis (*n*, %)	18 (8.37%)	15 (8.29%)	2 (16.67%)	1 (4.55%)	1.4979	0.473
Septic shock (*n*, %)	6 (2.80%)	6 (3.33%)	0	0	1.1660	0.558
Arthritis (*n*, %)	59 (27.31%)	54 (29.67%)	4 (33.33%)	1 (4.55%)	6.4724	0.039
Cellulitis (*n*, %)	30 (13.89%)	27 (14.84%)	3 (25.00%)	0	4.9234	0.085
Subperiosteal abscess (*n*, %)	35 (16.20%)	29 (15.93%)	5 (41.67%)	1 (4.55%)	7.9420	**0.019**
Muscular abscess (*n*, %)	26 (12.04%)	22 (12.09%)	2 (16.67%)	2 (9.09%)	0.4237	0.809
Deep vein thrombosis (*n*, %)	4 (1.85%)	3 (1.65%)	1 (8.33%)	0	3.2301	0.199
Fracture (*n*, %)	10 (4.63%)	7 (3.85%)	2 (16.67%)	1 (4.55%)	4.1912	0.123
Septic emboli (*n*, %)	2 (0.93%)	2 (1.10%)	0	0	0.3771	0.828
ICU admission (*n*, %)	10 (4.63%)	6 (3.30%)	3 (25.00%)	1 (4.55%)	12.0104	0.002

ICU: intensive care unit.

**Table 3:** White cells count, C-reactive protein and erythrocyte sedimentation rate values at diagnosis.

	Total (*n* = 205)	Other Type Osteomyelitis (*n* = 172)	Neonatal Osteomyelitis (*n* = 12)	Spondylodiscitis (*n* = 21)	Chi-Square	*p* Value
WBC at diagnosis (*n*, %)						
Not elevated	132 (64.39)	111 (64.53)	4 (33.33)	17 (77.27)	7.5617	0.023
Elevated	73 (35.61)	61 (35.47)	8 (66.67)	4 (19.05)		
Median value/mm3 (*n*, IQR)	10.000 (8.808–14.000)	10.125 (8.173–14.018)	12.835 (9.002–17.485)	8.220 (6.470–9.670)		0.0318
CRP at diagnosis (*n*, %)						
Negative	46 (22.12)	39 (22.29)	3 (25.00)	4 (22.12)	1.2970	0.862
Moderately elevated	86 (41.35)	70 (40.00)	5 (41.67)	11 (52.38)		
Markedly elevated	76 (36.54)	66 (37.71)	4 (33.33)	6 (28.57)		
Median value mg/dL (*n*,IQR)	2.62 (0.70–7.18)	2.63 (0.70–7.38)	4.48 (1.78–6.23)	1.94 (0.71–7.14)		0.884
ESR at diagnosis (*n*, %)						
Negative	23 (13.45)	20 (13.70)	3 (42.86)	0	8.5600	0.073
Moderately elevated	32 (18.71)	26 (17.81)	1 (14.29)	5 (27.78)		
Markedly elevated	116 (67.84)	100 (68.49)	3 (42.86)	13 (72.22)		
Median value mm/h (*n*, IQR)	45 (21.5–62)	44 (21–60)	13 (6–60)	53 (30–65)		0.318

IQR: interquartile range. WBC: white blood cells. WBC < 12,000/mm^3^: not elevated, WBC > 12,000/mm^3^: elevated. CRP: C-reactive protein. CRP < 0.5 mg/dL: negative; CRP 0.5–10 mg/dL: moderately elevated; CRP > 10 mg/dL: markedly elevated. ESR: erythrocytes sedimentation rate. ESR < 15 mm/h: negative; ESR 15–30 mm/h: moderately elevated; ESR > 30 mm/h: markedly elevated.

**Table 4:** Duration of antibiotic therapy.

	Total (*n* = 216)	Other Type Osteomyelitis (*n* = 182)	Neonatal Osteomyelitis (*n* = 12)	Spondilodyscitis (*n* = 22)	*p* Value
Intravenous therapy Days (IQR)	21 (15.5–28.5)	21 (15–27)	33 (27–44)	25.5 (19–32)	<0.001
Oral therapy Days (IQR)	15 (13–30)	15 (13–29)	15 (12–35)	20 (13–52)	0.198
Total Days (IQR)	37.5 (29–48)	37 (28–46)	48 (41–70)	48 (34–70)	<0.001

IQR: interquartile range.

**Table 5:** First line intravenous antibiotic therapy.

	Total (*n* = 216)	Other Type Osteomyelitis (*n* = 182)	Neonatal Osteomyelitis (*n* = 12)	Spondilodyscitis (*n* = 22)	Chi Square	*p* Value
antistaphylococcal penicillin + third generation cephalosporin	129 61.72%	112 63.64%	4 36.36%	13 59.09%	63.3552	**<0.001**
ampicillin + aminoglycoside	5 2.39%	1 0.57%	4 36.36%	0		
Clindamycin + third generation cephalosporin	9 4.31%	8 4.55%	0	1 4.55%		
glycopeptide + third generation cephalosporin	13 6.22%	12 6.82%	0	1 4.55%		
oxacillin monottherapy	11 5.26%	9 5.11%	0	2 9.09%		
cephalosporin monotherapy	6 2.87%	6 3.41%	0	0		
clindamycin monotherapy	4 1.91%	3 1.70%	0	1 4.55%		
Other	32 15.31%	25 14.20%	3 27.27%	4 18.18%		

**Table 6:** Switch to oral antibiotic regimens.

	Total (*n* = 216)	Other Type Osteomyelitis (*n* = 182)	Neonatal Osteomyelitis (*n* = 12)	Spondilodyscitis (*n* = 22)	Chi Square	*p* Value
Switch to oral therapy (*n*, %)						
Yes	176 (85.44%)	146 (84.39%)	10 (90.91%)	20 (90.91%)	0.9457	0.623
No	30 (14.56%)	27 (15.61%)	1 (9.09%)	2 (9.09%)		
Amoxicillin + clavulanate	95 (53.98%)	79 (54.11%)	9 (90.00%)	7 (35.00%)		
Flucloxacillin	8 (4.55%)	8 (5.48%)	0	0		
Clindamycin	11 (6.25%)	9 (6.16%)	0	2 (10.00%)		
trimethoprim/sulfamethoxazole + rifampicin	20 (11.36%)	5 (10.27%)	0	4 (25.00%)		
Amoxicillin + rifampicin	9 (5.11%)	5 (3.42%)	0	4 (20.00%)		
Linezolid	12 (6.82%)	10 (6.85%)	1 (10.00%)	1 (5.00%)		
Clidamycin + rifampicin	2 (1.14%)	2 (1.37%)	0	0		
Cephalosporin	7 (3.98%)	7 (4.79%)	0	0		
Other	12 (6.81%)	11 (6.25%)	0	1 (50.00%)		

**Table 7:** Summary of results.

	Other Type Osteomyelitis	Neonatal Osteomyelitis	Spondylodiscitis
Epidemiology		Among the different groups analysed, it is confirmed the higher incidence of multifocal osteomyelitis in neonatal group	
Symptomatology		Local symptoms prevail over systemic symptoms like fever: swelling is a prevalent sign of local inflammation	Pain and movement limitation prevail over other symptoms
Interval between onset of symptoms and start of antibiotic therapy			Longer latency time (12 days IQR 2–27) between onset of symptoms and start of antibiotic therapy, due to the lower expression of systemic symptom like fever
Inflammation markers at diagnosis	In most patients there is no leukocytosis at the time of diagnosis		In all patients, ESR was elevated at the time of diagnosis
Complicated osteomyelitis		Greater presence of osteoarthritis and subperiosteal abscesses and higher rate for intensive care admission than in the other groups.	
Instrumental examinations	MRI is confirmed as the gold standard test for the diagnosis of AHOM	US is able to provide information about the presence of subperiosteal abscesses and can be used in the diagnostic process. MRI remains the gold standard for diagnostic confirmation	
Therapy	Longer duration of intravenous antibiotic therapy than indicated in the ESPID guidelines	Longer duration of intravenous antibiotic therapy in neonatal and spondylodiscitis groups compared to the group of other types of osteomyelitis and in respect of what is indicated in the ESPID guidelines	
Pathogens	*S. aureus* is confirmed the first pathogen isolated in all three groups analysed, followed by *Streptococcus* and gram-bacteria as *P. aeruginosa* and *E.coli*		
Outcome and sequelae	Outcome without sequelae was confirmed in 93% of cases of treated patients.		

AHOM: acute haematogenous osteomyelitis.

## Data Availability

The datasets used and analysed during the current study are available from the corresponding author on request.
